# Diabetes Mellitus and Alzheimer’s Disease: The Protection of Epigallocatechin-3-gallate in Streptozotocin Injection-Induced Models

**DOI:** 10.3389/fphar.2017.00834

**Published:** 2017-11-16

**Authors:** Jin-Jing Jia, Xian-Si Zeng, Xin-Qiang Song, Peng-Peng Zhang, Lei Chen

**Affiliations:** ^1^College of Life Sciences, Xinyang Normal University, Xinyang, China; ^2^Institute for Conservation and Utilization of Agro-bioresources in Dabie Mountains, Xinyang Normal University, Xinyang, China; ^3^Henan Key Laboratory of Tea Biology, Xinyang Normal University, Xinyang, China

**Keywords:** diabetes, Alzheimer’s disease, streptozotocin, epigallocatechin-3-gallate, protection

## Abstract

Diabetes mellitus is considered as a risk factor of Alzheimer’s disease (AD), the front runner of neurodegenerative disorders. Streptozotocin (STZ) is a toxin for pancreatic β-cell, which can construct a model of insulin deficient diabetes through intraperitoneal or intravenous injection. A model generated by intracerebroventricular STZ (icv-STZ) also shows numerous aspects of sporadic AD. The protective roles of tea polyphenols epigallocatechin-3-gallate (EGCG) on both two diseases were researched by some scientists. This review highlights the link between diabetes and AD and recent studies on STZ injection-induced models, and also discusses the protection of EGCG to clarify its treatment in STZ-induced diabetes and AD.

## Introduction

Diabetes mellitus (DM) is a prevalent chronic endocrine diseases characterized by increased serum glucose concentrations, which is classified as type 1, type 2, gestational DM (GDM) and some other distinctive types. Type 1 diabetes mellitus, namely insulin-dependent DM (IDDM), is ordinarily caused by the autoimmune damage of pancreatic islet β-cells, resulting in the pancreas unable to synthetise and secrete insulin (Castano and Eisenbarth, 1990). Type 2 diabetes mellitus, formerly known as non-IDDM, is caused by a combination of inadequate insulin secretion and insulin resistance (Reaven, 1988). GDM is more similar to type 2 and attacks about 7% of pregnancies, usually remits after delivery, and composes a major risk factor for the development of type 2 diabetes later in life (Sacks et al., 2011). Other types of diabetes are of rarity. Metabolic impairments of such disease are a substantial cause of severe biochemical, molecular, and functional complications in many organs, consequently leading to progressive damage to the whole body.

Alzheimer’s disease (AD) is the most common one of neurodegenerative diseases and the main cause for dementia. AD is characteristically marked by progressive decline of cognitive functions and loss of learning and memory. The major pathological hallmarks are amyloid-β(Aβ) plaques and intracellular neurofibrillary tangles (NFT) and other molecular and biochemical abnormalities, including cell loss, impaired cellular metabolism, increased oxidative stress, and mitochondrial dysfunction (Mattson, 2004). AD is divided into early onset familial AD and late-onset sporadic AD. Familial AD constitutes no more than 1% of all AD patients, and the pathomechanism of most familial AD is related with the mutations of the proteins, such as Presenilin 1 (PS1), Presenilin 2 (PS2), and Aβ precursor protein (APP) (Bird, 2008). More than 99% of AD patients represent the so-called sporadic form, which is involved with various etiopathogenic mechanisms, such as brain trauma, inflammation, impairment of brain glucose metabolism, DM, and the gene dose of apolipoprotein E type 4 allele (Corder et al., 1993; Iqbal and Grundke-Iqbal, 2010).

Diabetes has been clinically considered as a risk factor of AD. Diabetes and AD are always the research focus and aporia of scientists. Streptozotocin (STZ)-treated rodents have become a reliable model to investigate the mechanisms and therapy of diabetes and AD. This article will review the relationship between diabetes and AD and the STZ-induced models. The roles of epigallocatechin-3-gallate (EGCG) in diabetes and AD were studied by some scientists, thus we also summarize the protection of EGCG in SZT injection-induced models.

## Relations Between Diabetes and AD

There is a close relationship between diabetes and AD. Some studies have detected the associations between metabolism and neuroplasticity at the cellular level and the systems level, where mitochondrial function, activation of insulin receptor, and expression of surface glucose transporter-3 (GLUT3) have all been linked with synaptic mechanisms for learning and memory. Proliferator-activated receptor γ co-activator-1α and mitochondrial biogenesis have important roles in the formation and maintenance of hippocampal dendritic spines and synapses (Cheng et al., 2012). Insulin receptor signaling regulates the formation of dendritic spine and the development excitatory synapse in hippocampal neurons through activation of the Rac1 and PI3K/Akt/mTOR signaling pathways (Lee et al., 2011). NMDA receptor-induced increase of cell surface GLUT3 represents a new signal pathway for control of energy supply during neuroactivity that is vital for preserving glucose homeostasis during neurotransmission (Ferreira et al., 2011). Diabetes have yet been clinically considered as a cause of cognitive impairment and dementia since Ott et al. (1996) reported that type 2 diabetes was associated with dementia in the Rotterdam study in 1996 and predicted that AD might be more frequent in senile DM patients administrated with insulin. Accumulated studies have shown that non-IDDM might be one risk factor of AD (Arvanitakis et al., 2004). Oxidative stress, mitochondrial dysfunction, impaired cellular metabolism, insulin resistance, inflammation and encephalatrophy are the common denominators in type 2 diabetes and AD (De Felice and Ferreira, 2014). Early brain abnormalities in AD are known as cerebral impaired glucose metabolism and insulin signaling (Yan et al., 2013; Chen et al., 2014).

### Glucose Metabolism

One ordinary abnormality of AD brain is the impairment of brain glucose uptake and metabolism, occurring many years before the first symptom appears, suggesting that the impairment of glucose uptake and metabolism may be mechanistically involved in AD or be a cause of neurodegeneration. Particularly, the impaired glucose utilization and relevant metabolic pathways are well-established findings and prominent in initial AD, analogous abnormalities of metabolism have been found in non-IDDM. Impairment of cerebral glucose utilization and energy metabolism arise very early in preliminary stages of cognitive decline (Cao et al., 2003), especially in sporadic AD which is characterized by a progressive exacerbation of both energy metabolism and cognition (Hoyer et al., 1991). It has been reported that the decrease in cerebral glucose utilization ranges from 10% to more than 40% in different levels of dementia (Kumar et al., 1991).

### Insulin Signaling

Besides reduced utilization of glucose, insulin receptor signal pathway is also severely damaged in the hippocampus of AD brain (Steen et al., 2005). Insulin signaling is related to massive cerebral functions, including cellular metabolism, synaptic plasticity and cognition (Banks et al., 2012). Recent evidence reveals that abnormal cerebral insulin signaling contributes to the development of AD (Ghasemi et al., 2014). Thus, enhancement of cerebral insulin signaling may be a brighter therapeutic strategy of AD. Intranasal administration of insulin has been shown to alleviates cognitive deficits and amyloid pathology in mice and humans (Benedict et al., 2004; Mao et al., 2016). The intranasal insulin delivery also has been proved to ameliorate memory in patients with mild cognitive impairment and AD (Craft et al., 2012).

### Oxidative Damage

In addition, the third major feature of pathophysiology in sporadic AD is oxidative stress, which can damage all endocellular biomacromolecules, consequentially resulting in neuronal dysfunction (Polidori and Mecocci, 2002). There is a strong relation among oxidative stress, metabolic syndrome, and AD. The high levels of circulating lipids and glucose imbalances amplify lipid peroxidation that gradually diminishes the antioxidant systems, causing high levels of oxidative metabolism that affects cell structure, leading to neuronal damage (Rojas-Gutierrez et al., 2017). What’s more, impairment of insulin signaling has already been associated with incremental mitochondrial dysfunction and oxidative stress in neurons (Hoyer and Lannert, 1999; Rains and Jain, 2011).

The pathological characteristics of sporadic AD are highly analogous to type 2 diabetes, therefore sporadic AD is considered as non-insulin-dependent diabetes mellitus of the brain (Hoyer, 2004). Numerous features of AD-like neurodegeneration can be experimentally produced together with increasing oxidative stress through impairing selectively insulin signaling, and support that AD represents a neuroendocrine disturbance related with brain-specific perturbations in insulin signaling mechanisms, namely type 3 diabetes (Lester-Coll et al., 2006; Song et al., 2017). The onomastion, “type 3 diabetes,” exactly reflects the fact that AD represents a form of diabetes that specially involves the brain and has biochemical and molecular characteristics that overlap with both type 1 and type 2 diabetes (de la Monte and Wands, 2008). Therefore, treatment with medicines, which promote glucose metabolism, enhance insulin signaling and reduce oxidative stress, might contribute to ameliorate the viability and function of brain neurons at the risk of AD neurodegeneration.

## STZ-Induced Models of Diabetes and AD

Streptozotocin, extracted from the *Streptomyces* species, is frequently used to induce model with robust deficits in neurogenesis (Ho et al., 2015), synaptic plasticity (Stranahan et al., 2008a), and cognition (Stranahan et al., 2008b) to study hippocampal plasticity in diabetes. Intraperitoneal (i.p.) injection of STZ is used to produce diabetes in rodents at wide range of doses (Baydas et al., 2003). Type 2 diabetes could be induced by an intraperitoneal injection of STZ. Rats were intraperitoneally injected with STZ (60 mg/kg) for 1 week and DM models were adopted only when blood glucose levels were above 16.7 mM on the third day after injection of STZ (Tian et al., 2016). In this model of diabetes, the impaired cognitive ability was evaluated by Morris water maze and decreased synaptic plasticity was monitored via examining the expression of brain derived neurotrophic factor (BDNF) and *N*-methyl-D-aspartate receptor (NMDAR1).

Intracerebroventricular injection of STZ (icv-STZ) by using stereotaxic technique is usually used to induce AD model at a dose of 3 mg/kg. The bregma coordinates are: -1.0 mm lateral, -0.3 mm posterior, -2.5 mm below (for 6 months old mice) (Chen et al., 2012, 2013) and +1.5 mm lateral, -0.8 mm posterior, -3.6 mm below (for 3 months old rat) (Guo et al., 2017). Icv-STZ provides a relevant animal model of chronic brain dysfunction that is characterized by progressive impairments in learning and memory, and cognition, an increase in Aβ-42, along with a permanent and ongoing cerebral energy deficit (Halawany et al., 2017). In this model, some glycolytic enzymatic activities were significantly reduced (Plaschke and Hoyer, 1993).

Thus it can be seen that either intraperitoneal or intracerebroventricular injection of STZ successfully induce models of diabetes and AD. This animal model may be useful for exploring the pathophysiological relationship between diabetes and AD and provides a new tool for development of effective therapy.

## Protection of EGCG in STZ-Induced Diabetes and AD

**FIGURE 1:**
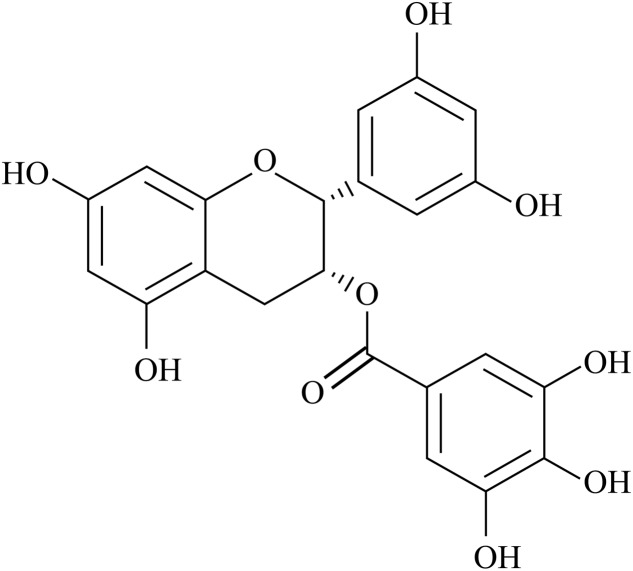
The chemical structure of EGCG.

Today there are millions of persons suffering from diabetes and AD and scientists never stop seeking the effective therapeutic drug. Green tea is very popular all over the world, especially in China. The green tea polyphenols are natural flavonoids that comprise various catechins, mainly epigallocatechin-3-gallate (EGCG) which is the most abundant antioxidant component and comprises approximately one third of green tea dry weight (Yang et al., 1998), with protective effects related to their antioxidant property (Sabu et al., 2002). The chemical structure of EGCG is illustrated in **Figure 1**. EGCG is a complex molecule formed by a flavanol core structure with a gallocatechol group and a gallate ester (Botten et al., 2015). These two gallocatechol rings confer the potent antioxidant and chelating properties to EGCG (Braicu et al., 2013). Each of the gallocatechol rings is able to directly capture reactive oxygen species from the environment with high efficiency (Nanjo et al., 1996). The pyrogallol group provides EGCG with strong metal-chelating ability, which allows it to bind transition metal ions acting as an antioxidant (Zhang et al., 2000). The galloyl group has also been associated with inhibitory effects on the microsomal enzyme system (Chen and Zhang, 2007) as well as with lipid lowering action (Kim et al., 2014). In the last two decades, scientists paid attention to the protection of EGCG on diabetes and AD due to its robust antioxidant property.

### Protection of EGCG in STZ-Induced Diabetes

Regular administration of green tea polyphenols, especially EGCG, have received considerable attention due to its metabolic effects for preventing metabolic diseases and type 2 diabetes (Wolfram, 2007). Numerous investigations have reported the effects of EGCG on the regulation of metabolic and brain function (Izzo et al., 2017; Mi et al., 2017; Yamamoto et al., 2017; Ye et al., 2017). Several studies suggest that the anti-diabetic effects of EGCG are probably due to suppressing appetite, modifying dietary fat emulsification in the gastrointestinal tract, inhibiting gastrointestinal lipolysis and reducing nutrient absorption (Raederstorff et al., 2003; Shishikura et al., 2006).

#### Antioxidant Effects

The protection of EGCG on STZ-induced diabetes mainly focuses on its antioxidant activity (**Table 1**). An elevation of malondialdehyde (MDA) and an decrease of superoxide dismutase (SOD) activity were observed in aortic tissue of STZ-induced diabetic rats. Chronic EGCG treatment could impede the abnormal functional changes of vascular reactivity via restoring the SOD activity and further inhibiting oxidative stress (NO) (Roghani and Baluchnejadmojarad, 2009). EGCG administration (p.o.) also reversed the incremental MDA concentration and the decreased SOD activity in liver (Roghani and Baluchnejadmojarad, 2010). Treatment with EGCG in STZ-induced diabetes of mice ameliorated the decrease of pancreatic islet mass and repressed the increase of blood glucose levels, as well as the expression of inducible NOS (iNOS) which is in accordance with the above study (Song et al., 2003). The alleviation of EGCG on STZ-induced diabetic neuropathic hyperalgesia is also involved in oxidative stress (Baluchnejadmojarad and Roghani, 2012). The findings suggested the therapeutic potential of EGCG in diabetic hyperalgesia through the inhibition of oxidative stress. Roposo et al. (2015) reported that EGCG intake (2 g/L in water) could prevent STZ-induced diabetic neuropathic pain via normalizing the increase of 8-hydroxy-2′-deoxyguanosine (8-OHdG) though the neurobiological mechanisms is still unknown. Mostafa et al. (2013) demonstrated that a lower concentration of EGCG (7.6 mg/L in water) significantly increased glutathione peroxidase (GPx) and decreased the cavernous MDA compared with diabetic rats. EGCG also showed therapeutic potential on renal damage via suppressing hyperglycemia, proteinuria, and lipid peroxidation in diabetic nephropathy model rats treated with STZ (Yamabe et al., 2006). EGCG reduced the elevated MDA levels and attenuated oxidative stress, and reversed the expression of manganese superoxide dismutase (MnSOD), attenuates myocardial injury induced by ischemia/reperfusion in STZ injection (i.p.)-induced diabetic rats (Wu et al., 2017).

**Table 1:** The anti-oxidant effects of EGCG in STZ-induced Diabetes and AD.

Disease	Species	STZ Injection Pattern	EGCG dose	Protective Mechanism	Reference
Diabetes	Rats	i.p.	25 mg/kg	↑SOD,↓MDA, ↓NO	Roghani and Baluchnejadmojarad, 2009
	Rats	i.p.	60 mg/kg	↑SOD,↓MDA	Roghani and Baluchnejadmojarad, 2010
	Mice	i.p.	100 mg/kg	↑pancreatic islet mass, ↓iNOS	Song et al., 2003
	Rats	i.p.	40 mg/kg	↑SOD,↓nitrite,	Baluchnejadmojarad and Roghani, 2012
	Rats	i.p.	2 g/L (in water)	↓oxidative stress (8-OHdG)	Raposo et al., 2015
	Rats	i.p.	7.6 mg/L (in water)	↓MDA, ↑glutathione peroxidase	Mostafa et al., 2013
	Rats	i.p.	100 mg/kg	↓oxidative stress, ↓MDA, ↑MnSOD	Wu et al., 2017
	Rats	i.p.	50–100 mg/kg	↓oxidative stress	Yamabe et al., 2006
	Mice	i.p.	100 mg/kg	↓oxidative damage, ↑Nrf2	Pan et al., 2017; Sun et al., 2017
AD	Rats	i.p.	40 mg/kg	↑glutathione peroxidase, ↓reactive oxygen species, ↓NO production	Baluchnejadmojarad and Roghani, 2011
	Rats	icv.	10 mg/kg	↑learning and memory, ↓malondialdehyde, ↓NO	Biasibetti et al., 2013


*(↑) Increase, (↓) Decrease for STZ + EGCG group vs. STZ group.*

Nuclear factor erythroid 2-related factor (Nrf-2), a primary antioxidant transcription factor, shows a reduced expression in type 2 diabetes mellitus patients. Impairments in this antioxidant system leads to type 2 diabetes mellitus-associated inflammatory (Vaamonde-Garcia et al., 2017). A recent study showed that EGCG prevent mice from diabetic nephropathy through upregulating the expression of Nrf2 and further inhibiting diabetes-induced renal oxidative damage (Sun et al., 2017). In addition, EGCG preserved testicular weight and spermatozoa count, and attenuated oxidative damage via activating Nrf2 expression and function in STZ-induced diabetic mice (Pan et al., 2017).

#### Other Effects

Some studies have showed that EGCG has glucose-lowering, lipid-lowering anti-inflammatory, and anti-apoptosis effects in STZ-induced diabetes models (**Table 2**). STZ (i.p.) treated mice indicated an increase in levels of blood glucose, which was significantly reversed with EGCG treatment (Song et al., 2003; Yoon et al., 2014). EGCG has the potential to control hyperglycemia by attenuating advanced glycation end products formation (Sampath et al., 2017). Yamabe et al. (2006) found that EGCG had a positive effect on serum glucose and lipid metabolic abnormalities in STZ injection-induced diabetes. EGCG intake from drinking water also showed a glucose-lowering effect in STZ injection-induced diabetes rats (Mostafa et al., 2013). Lipophilic EGCG derivative also showed antidiabetic activities, including reducing the plasma glucose and promoting lipid metabolism, in STZ-induced diabetic rats (Li et al., 2007). In STZ-induced diabetic mice, EGCG attenuated testicular inflammation, endoplasmic reticulum stress and apoptotic cell death (Pan et al., 2017). Oral EGCG for 30 days after DM production notably inhibited the increase of pro-inflammatory cytokines (IL-1 β, IL-6 and TNF-α) in serum, suggesting its anti-inflammatory potential (Othman et al., 2017). Mohan et al. (2017) reported that EGCG Supplementation resisted STZ-induced diabetic nephropathy in rats via inhibiting mitochondrial apoptosis pathway (downregulating Bax/Bcl-2, caspase-3). EGCG inhibited the apoptosis via reducing the elevated lactate dehydrogenase (LDH) and attenuated myocardial injury induced by ischemia/reperfusion in STZ diabetic rats (Wu et al., 2017) (**Table 2**).

**Table 2:** The glucose-lowering, anti-inflammatory, anti-apoptosis and lipid-lowering effects of EGCG in STZ-induced Diabetes.

Species	STZ Injection Pattern	EGCG dose	Effects	Reference
Mice	i.p.	100 mg/kg	↓blood glucose	Song et al., 2003; Yoon et al., 2014
Rats	i.p.	50–100 mg/kg	↓serum glucose, ↓lipid peroxidation	Yamabe et al., 2006
Rats	i.p.	7.6 mg/L (in water)	↓serum glucose	Mostafa et al., 2013
Rats	i.p.	50 mg/kg	↓plasma glucose, ↑lipid metabolism	Li et al., 2007
Mice	i.p.	100 mg/kg	↓inflammation	Sun et al., 2017
Mice	i.p.	100 mg/kg	↓inflammation, ↓endoplasmic reticulum stress, ↓apoptosis	Pan et al., 2017
Rats	i.p.	2 mg/kg	↓inflammation, ↓apoptosis	Othman et al., 2017
Rats	i.p.	100 mg/kg	↓apoptosis	Mohan et al., 2017
Rats	i.p.	100 mg/kg	↓apoptosis	Wu et al., 2017


*(↑) Increase, (↓) Decrease for STZ + EGCG group vs. STZ group.*

### Protection of EGCG in STZ-Induced AD

Scientists have revealed that EGCG plays various roles in AD, including behavior, physiology and pathology. It have been reported that many articles regards the potential effects of EGCG in the treatment of AD (Mandel et al., 2007, 2008; Afzal et al., 2015; Walker et al., 2015; Cascella et al., 2017; Xicota et al., 2017). EGCG ameliorates learning and memory deficits by adjusting the balance of TrkA/p75NTR signaling (Liu et al., 2014). EGCG could not only attenuate Aβ generation (Dasilva et al., 2010; Zhang et al., 2017), but also facilitate its degradation by increasing neprilysin secretion from astrocytes through activation of ERK and PI3K pathways (Yamamoto et al., 2017). Therefore, EGCG could inhibit the oxidative stress in AD and decrease the apoptosis of neurons. Protection of EGCG in rodent models of AD has currently obtained much research attention. Incremental number of investigations reported that EGCG has neuroprotection in AD models, for example, Aβ or lipopolysaccharide-injected mice, and transgenic mice overexpressing Aβ (Rezai-Zadeh et al., 2005, 2008; Lee J.W. et al., 2009; Lee Y.K. et al., 2009). Although the STZ-induced model is widely used to investigate the mechanisms of sporadic AD and the develop some useful medicines for AD, there are few literatures about the protective effects of EGCG in STZ-induced AD. So far as I know, sporadic studies focused on the protection of EGCG in STZ-induced AD. Baluchnejadmojarad and Roghani (2011) showed that chronic green tea EGCG treatment could dose-dependently ameliorate learning and memory deficits in intraperitoneal injection of STZ-induced rats through attenuation of oxidative stress and modulation of NO which is accordance with others’ results. Biasibetti et al. (2013) indicated that EGCG administration could ameliorate learning and memory and regulate oxidative stress, in which glutathione peroxidase activity, NO production and reactive oxygen species content were reversed in a icv-STZ-induced rat model of dementia (**Table 1**). However, EGCG was not able to restore the reduced glucose uptake by icv-STZ. The different effect of EGCG on glucose may due to the diverse doses, species or procedure schedules in the experiments.

## Conclusion and Expectation

Diabetes and AD are two of incurable chronic diseases. There are the common denominators in type 2 diabetes and AD, such as energy metabolic dysfunction, inflammation, insulin resistance, mitochondrial dysfunction, and oxidative stress. It is reasonable that diabetes is considered as risk factor of AD. Injection of STZ (i.p. or icv) could induce model of diabetes and sporadic AD, which is beneficial to research the relationship between the two and seek effective therapeutic strategies. Based on the above review, it has been proved that EGCG has protective role in diabetes and AD, but the investigations are actually limited and restricted to antioxidant field. In my submission, it should be well performed to study the effect of EGCG treatment on glucose metabolism and insulin signaling, as well as the corresponding mechanisms, which may contribute to easily understand the pathogeny of diabetes and AD and the protective role, and promote its clinical application.

## Author Contributions

J-JJ and X-SZ wrote the original paper. X-QS searched the related literatures. X-SZ, P-PZ, and LC revised the paper.

## Conflict of Interest Statement

The authors declare that the research was conducted in the absence of any commercial or financial relationships that could be construed as a potential conflict of interest.
